# Constitutive basis of root system architecture: uncovering a promising trait for breeding nutrient- and drought-resilient crops

**DOI:** 10.1007/s42994-023-00112-w

**Published:** 2023-09-15

**Authors:** Zhigang Liu, Tongfei Qin, Michaella Atienza, Yang Zhao, Hanh Nguyen, Huajin Sheng, Toluwase Olukayode, Hao Song, Karim Panjvani, Jurandir Magalhaes, William J. Lucas, Leon V. Kochian

**Affiliations:** 1 Global Institute for Food Security, University of Saskatchewan, Saskatoon, SK S7N 4L8 Canada; 2 Department of Computer Science, University of Saskatchewan, Saskatoon, SK S7N 5C9 Canada; 3 Embrapa Maize and Sorghum, Brazilian Agricultural Research Corporation, Sete Lagoas, MG 35701-970 Brazil; 4 Department of Plant Biology, College of Biological Sciences, University of California, Davis, CA 95616 USA

**Keywords:** Constitutive root system architecture, Abiotic stress, Nutrient efficiency, Drought resilience, Plant breeding

## Abstract

Root system architecture (RSA) plays a pivotal role in efficient uptake of essential nutrients, such as phosphorous (P), nitrogen (N), and water. In soils with heterogeneous nutrient distribution, root plasticity can optimize acquisition and plant growth. Here, we present evidence that a constitutive RSA can confer benefits for sorghum grown under both sufficient and limiting growth conditions. Our studies, using P efficient SC103 and inefficient BTx635 sorghum cultivars, identified significant differences in root traits, with SC103 developing a larger root system with more and longer lateral roots, and enhanced shoot biomass, under both nutrient sufficient and deficient conditions. In addition to this constitutive attribute, under P deficiency, both cultivars exhibited an initial increase in lateral root development; however, SC103 still maintained the larger root biomass. Although N deficiency and drought stress inhibited both root and shoot growth, for both sorghum cultivars, SC103 again maintained the better performance. These findings reveal that SC103, a P efficient sorghum cultivar, also exhibited enhanced growth performance under N deficiency and drought. Our results provide evidence that this constitutive nature of RSA can provide an avenue for breeding nutrient- and drought-resilient crops.

**Supplementary Information:**

The online version contains supplementary material available at 10.1007/s42994-023-00112-w.

## Introduction

It has been estimated that, by 2050, the global human population will reach 10 billion (Hickey et al. 2019), hence, global agricultural production must increase to meet this demand. In this regard, evidence available for maize, rice, wheat, and soybean, which produce the majority of global agricultural calories, indicates from current progress in yield enhancement that there is a significant need for novel crop improvement strategies (Gao 2021; Ray et al. 2013). Due to abiotic stresses, including drought, flooding, extreme temperatures, salinity, and acid soil conditions, and limiting nutrient availability and cost, both crop growth and yield can be greatly impacted (Halford et al. 2015; Tyczewska et al. 2018). This issue is of great concern and requires immediate attention to ensure sustainable food production in the face of these environmental challenges.

Until recently, the underground component of crops, namely their root systems, have received little attention regarding crop improvement. Given the important functions of the root system, including water and nutrient acquisition, and plant anchorage, recent studies have begun to explore the functional components of root system architecture (Gabay et al. 2023; Hodge et al. 2009; Ryan et al. 2016; Zheng et al. 2023). In monocots, the root system contains seminal (primary), lateral and crown roots, whereas in dicots, a primary root (tap root) system gives rise to multiple orders of lateral roots (Meister et al. 2014). Root system architecture (RSA), the spatial configuration of the plant root system, plays a pivotal role in efficient uptake of essential nutrients (e.g., nitrogen [N], phosphorous [P], and potassium [K]) and water (Marschner 2011).

**Fig. 1:**
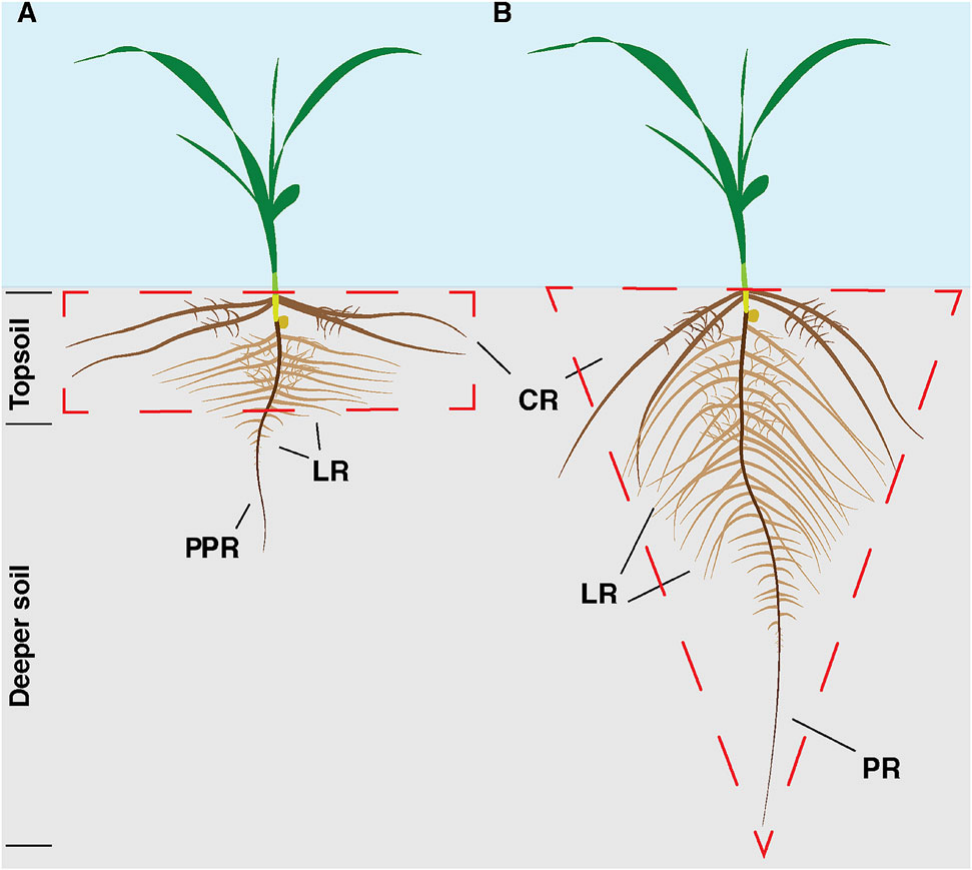
Schematic representation of root system architectures representing topsoil based and deep rooting systems. **A** Plants that develop a shallow root system localized within the topsoil (broken rectangle). **B** Plants that develop an extended, deeper, root system (broken inverted triangle). *CR* crown root, *LR* lateral root, *PR* primary root

In agricultural soils, the spatial availability of these essential components for growth are often heterogenous in nature. Thus, soil characteristics must be integrated into breeding programs to achieve optimal nutrient acquisition for enhanced yield performance (Lynch 2022; Tracy et al. 2020). Numerous studies have focused on the role of RSA in efficient acquisition of the most diffusion-limited mineral nutrient, phosphorous (as phosphate [Pi]) (Liu 2021; Mohammed et al. 2022). Here, in acid soils, Pi is tightly bound to clay minerals, within the topsoil, resulting in a marked vertical Pi gradient. These soil characteristics have driven researchers to focus on selection of P efficient lines, in which a majority of the RSA is located within the topsoil (Liang et al. 2010; Rangarajan et al. 2018; Sun et al. 2018; Zhao et al. 2004) (Fig. 1A).

Under acid soil conditions, the mobile nutrients, including N and K, are distributed throughout the soil profile. Thus, in breeding P efficient lines, consideration is needed in terms of acquisition of these mobile nutrients. In this regard, a topsoil RSA would likely require highly efficient N and K transport systems for capture during fertilizer application. An alternative breeding approach might utilize an RSA in which the roots are more uniformly distributed throughout the soil profile (Fig. 1B).

Sorghum breeding programs have developed Pi efficient lines optimized for crop performance on acid soils. For example, Hufnagel et al. (2014) identified P efficient and inefficient lines based on screening of a 243-line sorghum association panel for grain yield, when grown on a low P tropical soil. In this study, significant genetic variation in P efficiency was identified. Also, by using candidate gene genome-wide association study (GWAS), several *PSTOL1* (*Phosphorous Starvation Tolerance 1*) genes were discovered. Equivalent *PSTOL1* genes were earlier identified in rice, as P efficiency genes (Gamuyao et al. 2012), in which breeding for this trait gave rise to larger and deeper root systems. These RSAs clearly did not concentrate a significant fraction of their root within the upper (topsoil) regions of the soil profile.

To further explore the relationship between RSA and yield performance, under field conditions with limiting soil P availability, in the current study, we utilized two sorghum lines, SC103 and BTx635, having significant differences in grain yield; BTx635 was 1.6 tons ha^−1^, whereas SC103 was 2.8 tons ha^−1^—a yield increase of 75% (Hufnagel et al. 2014). Based on the SC103 yield enhancement, we conducted an extensive study of their RSAs to ascertain whether the distribution of their roots conformed to either the topsoil pattern (Fig. 1A) or a broader and deeper distribution (Fig. 1B). Of special note, our findings failed to support the topsoil model, in that SC103 exhibited an “inverted triangle” RSA like that shown in Fig. 1B. Importantly, SC103 had enhanced performance under both full nutrient and limiting P, N, and drought conditions. Additionally, a similar RSA pattern was identified for BTx635, but overall, this root system was significantly smaller, under these same growth conditions for N, P, and water. These studies highlight the requirement for a coordination between RSA studies and crop breeding programs.

## Materials and methods

### Plant materials

Two sorghum (*Sorghum bicolor*) cultivars, SC103 and BTx635, were used in this study. These cultivars represent widely employed sorghum breeding lines in the United States and Brazil (Casa et al. 2008). Further, Hufnagel et al. (2014) demonstrated that SC103 had higher grain yields, compared to BTx635, when grown under on low P soil with high Pi soil fixation, reflecting limiting soil P availability. These studies established SC103 and BTx635 as relatively P efficient and inefficient, respectively.

### Plant growth for 2D root system architecture (RSA) assays

Seeds were surface-sterilized in 6% (v/v) sodium hypochlorite for 20 min, rinsed with 18 Mega-ohm water (MΩ), and germinated, in darkness, for 4 days at 27 °C on moist germination paper (Anchor Paper, St. Paul, MN, USA). Uniform seedlings were transferred, depending on the specific experiment, to hydroponic solution, silica sand or Sunshine #1 potting mix (Sun Gro Horticulture, Inc.) as the appropriate growth system for RSA studies. Plants were grown in a walk-in growth chamber with controlled climate conditions of 16 h/8 h (light/dark), 27 °C/22 °C (day/night) temperature, a light intensity of approx. 350 µmol m^−2^ s^−1^, at canopy height, and 40–60% relative humidity.

After germination, uniform sorghum seedlings were transplanted to 100 L polypropylene containers and grown hydroponically in a specially designed pouch system, as described by (Gladman et al. 2022). Plants were supplied with a nutrient solution consisting of macronutrients (at mM levels): 3.5 Ca(NO_3_)_2_, 1.3 NH_4_NO_3_, 0.58 K_2_SO_4_, 0.58 KCl, 0.56 KNO_3_, 0.85 MgSO_4_; and micronutrients (at μM levels): 2.5 H_3_BO_3_, 9.1 MnCl_2_, 0.6 CuSO_4_, 2.4 ZnSO_4_, 0.8 Na_2_ MoO_4_, 100 Fe-HEDTA. Nutrient solution phosphate (Pi) concentrations were 0, 2.5, 10 and 200 µM. Experiments were first conducted to establish the appropriate Pi concentrations for severe plant P deficiency (0 and 2.5 μM), moderate Pi deficiency (10 μM) and sufficient plant Pi status (200 μM): the quantitative indicators of P deficiency are a strong inhibition of shoot growth and a moderate stimulation of root growth, resulting in an increase in root:shoot ratio (Cakmak et al. 1994; Ericsson 1995; Chiera et al. 2002). The Pi concentrations were obtained by adjusting KH_2_PO_4_ and KCl was added to maintain the nutrient solution [K] concentrations close to at 3.11 mM, with 0.2 mM being added as KH_2_PO_4_. To assess the effects of N deficiency on plant RSA, seedlings of each cultivar were grown hydroponically for 10 days in a pouch system, with 4000 µM nitrogen as sufficient N (SN) and 400 µM as low N (LN) conditions. As N was added as Ca(NO_3_)_2_, to maintain the same Ca^2+^ concentration in the LN media, as used in the SN media, 900 µM CaCl_2_ was supplied in LN media. The nutrient solution was aerated continuously and renewed every 3 days. The solution pH value was maintained at 5.7.

In longer term experiments where sorghum was grown in pots, seedlings of the sorghum cultivars SC103 and BTx635 were transferred to plastic pots (25 cm diameter, and 20 cm depth) containing silica sand and nutrient solutions of different [Pi] were added to the top of the pots. The plants were grown for 28 days, using the above-described nutrient solutions, except that the Pi concentration used for control (CK) and low Pi (LP) conditions were adjusted to 500 and 75 µM, respectively, and the N concentration used for LN media was reduced to 600 µM. For experiments conducted with plants grown in silica sand, the appropriate concentrations of N and P for sufficiency and deficiency were determined empirically, by measuring and comparing shoot biomass for plants grown on LN and SN nutrient solution. For plants grown in sand watered with LP and SP solutions, shoot growth and root traits were evaluated as described for P deficiency in hydroponically grown plants in 2D pouches. Given that nutrient availability in silica sand is restricted to the pores within the sand, the nutrient concentrations in the sand experiments were adjusted to ensure sufficient or deficient levels. For LP, a concentration of 75 μM was determined to be moderately P deficient, whereas moderate P deficiency symptoms developed in plants grown in hydroponic media with 10 μM Pi. Regarding LN, 600 μM N generated moderately N deficient sand-grown plants, whereas 400 μM N in hydroponic media resulted in moderate N deficiency symptoms.

To evaluate drought performance of the sorghum cultivars, plants were grown in plastic pots, as above, containing 1.2 kg Sunshine #1 potting mix. Plants were fertilized biweekly using water-soluble fertilizer (Master Plant-Prod, Inc.), with a standard N–P–K (20–20–20) treatment. Pot water content was measured using an HydroSense II Handheld Soil Moisture Sensor (Campbell Scientific, Inc.), based on the volumetric water content (VWC) for porous media. Pots were initially filled with 18 MΩ water and then allowed to drain to establish soil ‘field capacity’. Seedlings were then transplanted, with one seedling per pot, and 200 mL of 18 mΩ water (corresponding to near-full field capacity) was added, daily, for the first 14 days.

After the initial two-week growth period, plants were divided into two treatment groups: well-watered (WW) and water stressed (WS). The WW plants continued to receive 200 mL of 18 MΩ water, daily, per pot, to maintain the water content near the full field capacity. For the WS plants water was withheld, and fourteen days later leaf wilting was observed, coincident with a measured potting mix water content of 7.5% VWC. Plants were subsequently removed from the pots and their root systems were soaked in water to remove potting mix, and then thoroughly cleaned by washing using low water pressure. Finally, RSA measurements were determined (Clark et al. 2013) and then roots and shoots were separated, following drying for biomass determination.

### Root architecture measurements

Phenotyping experiments, using a hydroponic pouch system, utilized a Nikon D7200 DSLR camera with a 50 mm lens and a 2D root imaging platform. Raw images were collected and stored in a Plant Root Imaging and Data Acquisition (PRIDA) program, and then extracted as TIFF files for further image processing and root trait computation (Gladman et al. 2022). For the phenotyping experiments performed in silica sand and potting mix, root systems were cleaned after harvest, arranged to minimize any root overlap, and then a 2D root imaging platform was used to acquire and store data. Data from phenotyping experiments were then subjected to both commercial and publicly available software packages for root trait computation. WinRHIZO software (Regents Instruments, Inc.) was used to quantify root growth and topology traits, and GiA Roots (Galkovskyi et al. 2012) was used to quantify 2D root architecture traits. Root architecture traits, derived from the hydroponic pouch system, included primary root length (cm), root system width (cm), and convex hull area (cm^2^), were assessed. Root morphology traits, derived from the hydroponic pouch system included average root diameter (cm), total root system surface area (cm^2^), total root system length (cm), and total root system volume (cm^3^). Root morphology traits, derived from experiments in which plants were grown in silica sand or potting mix, included total root system length (cm), root system surface area (cm^2^), and total root system volume (cm^3^), were assessed.

### Biomass, N and Pi measurements

After root architecture determinations, plants were dissected into shoots and roots, and both parts were heated at 105 °C for 30 min and then oven-dried at 65 °C for 72 h. The dry samples were then weighed to obtain shoot dry weight (SDW), root dry weight (RDW), and the root:shoot ratio (R/S). After weight measurements, the root and shoot samples were ground and digested, using a solution of concentrated sulfuric acid and selenium at 330 °C. Total P and N concentrations were then determined, using a Skalar SAN-plus segmented flow analyzer (Skalar Analytical BV, Breda, The Netherlands), following the manufacturer’s protocols.

### Root sectioning and imaging

Sorghum seedlings were grown hydroponically for 10 days in 20 L polypropylene containers in the same nutrient solution, as described above, under sufficient Pi (SP; 200 µM) or low Pi (LP; 2.5 µM) conditions. Growth protocols were as described above. Primary and crown roots were excised at 5 cm from the root-stem junction and at 5 cm from the root tip, respectively. Lateral roots were excised at 5 cm from the primary-lateral root junction and 5 cm from the lateral root tip. All root samples collected were ~ 5 cm in length. Root samples were placed into 3D-printed polylactic acid molds for embedding (Atkinson and Wells 2017). Root tissues were fixed in 5% (w/v) agarose, and after agarose solidification, blocks were trimmed and sectioned, at 100 µm, using a Leica VT1000S (Vibrating blade microtome, Nussloch, Germany). Transverse sections were examined using a Leica Thunder microscope system (Wetzlar, Germany). Images were collected using 10 × or 20 × objectives, depending upon the tissue size, and the background noise was removed from images using Adobe Photoshop version 23.5 (Adobe Systems). Root cross-sectional area (SCA) and aerenchyma area (AA) were measured, via pixel-counting, using ImageJ software (https://imagej.nih.gov/ij/). Proportion of root cross sections occupied by aerenchyma was calculated using AA divided by SCA. Sample data were normalized, based on a previously described method (Burton et al. 2015).

### Statistical analysis

All statistical analyses were performed using the R software package (Team 2013). Two-way analysis of variance (ANOVA) was used to test for significant differences between treatments, cultivars, and treatment × cultivar interactions. Significant differences between means were analyzed by independent Student’s *t* test or Tukey’s honest significant difference (HSD) tests, where appropriate. ANOVA was performed using the “Anova” function, as implemented in the “car” package (Fox et al. 2012). Tukey’s HSD tests were performed using the “Tukey HSD” function in R. Additional visualization of the data was performed using the “ggplot2” package (Wickham 2016).

## Results

### Sorghum SC103 establishes a larger root system under both sufficient and deficient Pi conditions

**Fig. 2:**
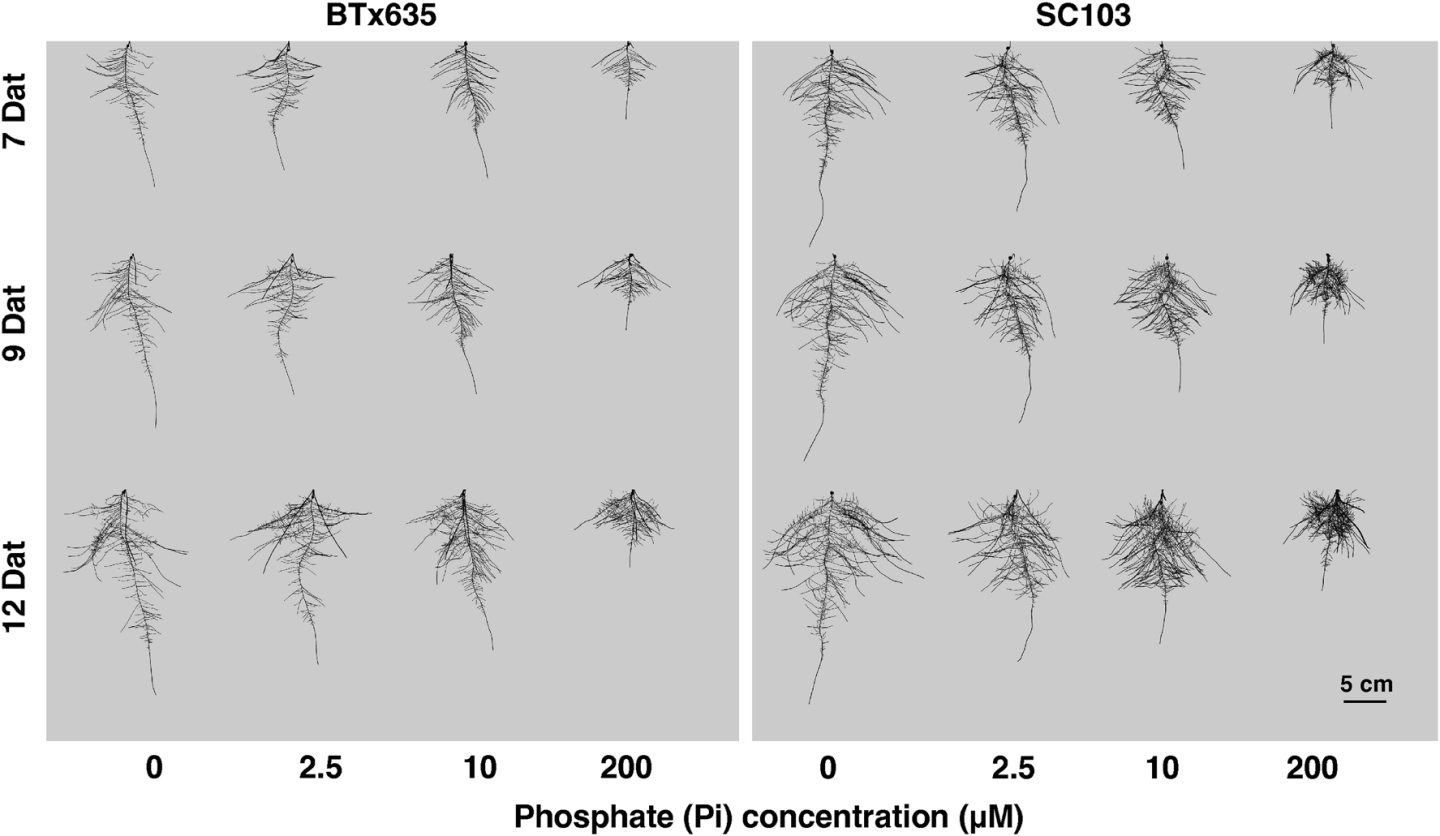
Response of sorghum root systems (lines SC103 and BTx635) grown under 200 μM Pi (sufficient P), and 10, 2.5 and 0 μM Pi (low P), for 7, 9 and 12 days after transplanting (Dat). A hydroponic pouch system was employed for these assays, and representative images are shown

**Fig. 3:**
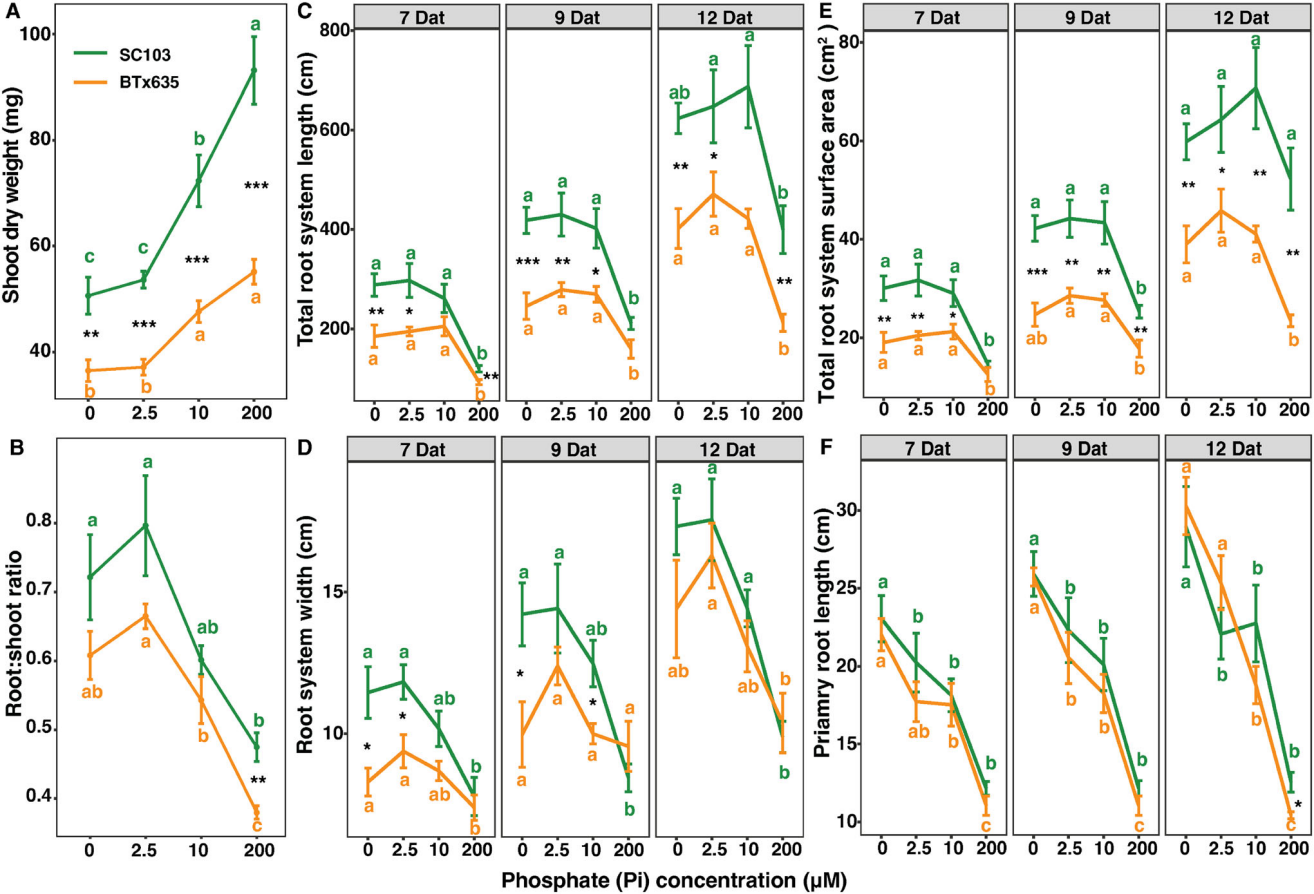
Root system architecture of sorghum lines SC103 and BTx635, grown in a hydroponic pouch system, under the indicated phosphate (Pi) concentrations. Panels represent shoot dry weight (**A**), root:shoot ratio (**B**), total root system length (**C**), root system width (**D**), total root system surface area (**E**), and primary root length (**F**), analyzed at 7, 9 and 12 Dat. Data shown are means ± SE (n = 6). Asterisks indicate significant differences between the genotypes, under the same Pi concentrations, as determined by the Student’s t test. For these assays, significance differences are indicated as follows: *P < 0.05; **P < 0.01; ***P < 0.001. Different lowercase letters indicate significant differences (*P* < 0.05) among the four Pi concentrations, in the same genotype, as determined by Tukey’s HSD tests

Sorghum seedlings were grown in a hydroponic pouch system for 7, 9, and 12 days after transplanting (Dat). This nutrient delivery system was used to provide Pi distribution to the root systems of these sorghum cultivars. Both cultivars exhibited significantly larger root systems in response to Pi deficiency (Fig. 2). However, SC103 also exhibited a much larger root system than BTx635 under Pi sufficient conditions. Furthermore, the P efficient SC103 exhibited 69 and 110% greater SDW and RDW, respectively, compared with the P inefficient BTx635, under Pi sufficient conditions (200 µM). As expected, a significant reduction in SDW was observed in both SC103 and BTx635, in response to Pi stress. However, SDW in SC103 was significantly greater than in BTx635, under all levels of the imposed Pi stress (Fig. 3A). An increase in the root:shoot ratio (R/S) is commonly observed under Pi deficient conditions, either by a reduction in shoot growth or an increase in root growth, or both (Cakmak et al. 1994; Ericsson 1995; Chiera et al. 2002). Under Pi stress, R/S ratio was increased by 27% (10 µM), 68% (2.5 µM) and 52% (0 µM) in SC103, whereas an increase of 43% (10 µM), 75% (2.5 µM) and 60% (0 µM) was observed in BTx635, compared with sufficient Pi conditions (Fig. 3B). The P deficiency severity was also increased by growth on 10, 2.5 and 0 µM Pi; here, a stronger inhibition of shoot growth and increased stimulation in lateral root growth was observed as P deficiency became more severe (Fig. 3A and Supplementary Table 1). This resulted in increases in R/S ratios under increasing P deficiency, which, as noted above, has been well-established in previous studies (Cakmak et al. 1994; Ericsson 1995; Chiera et al. 2002). Collectively, these findings support the notion that SC103 has a genetic component that imparts a capacity for establishing a larger root system under both sufficient and deficient Pi conditions.

### Sorghum SC103 developed a larger RSA compared with BTx635

Traits associated with RSA, including primary root length, total root system length, total root system surface area, root system width (Fig. 3), and average root diameter, total root system volume, and convex hull area (total area occupied by the root system) (Supplementary Table 1), were assessed. Under sufficient Pi conditions, total root system length (88%), root system surface area (117%), total root system volume (157%), primary root length (21%), and convex hull area (60%) were greater in SC103, compared with BTx635, at 12 Dat (Fig. 3C–F; Supplementary Table 1). Under Pi deficient conditions, total root system length (55%), total root system surface area (53%), and total root system volume (52%) were greater in SC103, compared with BTx635, at 12 Dat (Fig. 3C–F; Supplementary Table 1).

**Fig. 4:**
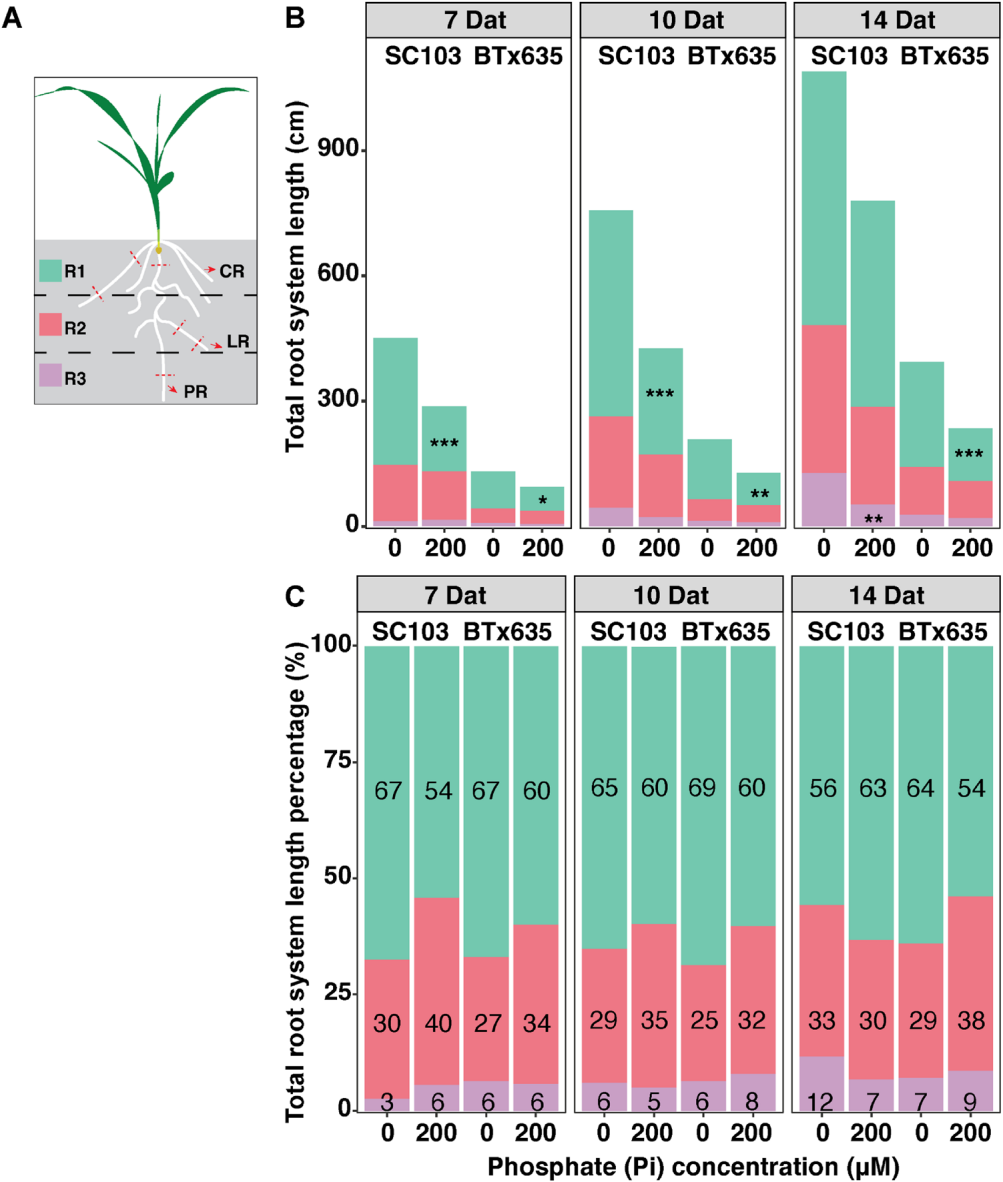
Root system architecture profiles for SC103 and BTx635 grown in a hydroponic pouch system, under sufficient Pi (200 μM) and deficient Pi (0 μM) conditions, for 7, 10 and 14 Dat. **A** Root systems were divided into three equal regions, R1, R2 and R3, based on the root system depth. **B** Total root system length in each region. **C** Percentage of total root system length in each of the three regions. Asterisks indicate significant differences between the various Pi concentrations, in the same cultivar, in the same region, as determined by Student’s t test: for these assays, significance differences are indicated as follows: *P < 0.05; **P < 0.01; ***P < 0.001. *CR* crown root, *PR* primary root, *LR* lateral root. Red dotted lines represent the locations where root sections were excised for cross-sectional analyses (see Figs. 5A, E, 6A, E, and Supplementary Fig. 2)

As demonstrated by the data presented in Fig. 3 and evident from the images in Fig. 2 showcasing the root systems of the two cultivars, grown for 7, 9, and 12 Dat at varying growth solution Pi concentrations, it is apparent that increasing P deficiency leads to greater stimulation of lateral and tap root growth, compared to plants grown under sufficient P conditions (200 μM Pi). Furthermore, investigating the root system vertical distribution can be beneficial for an understanding of the pattern of root Pi acquisition. For our study, the sorghum root system was divided into three equally-spaced regions, based on root system depth (Fig. 4A). In the top region (R1), we observed that SC103 had a significantly higher total root system length compared with BTx635 at all time points, under sufficient and deficient Pi conditions. Importantly, under Pi stress, total root system length was increased significantly at all time points in SC103 and BTx635 in region R1 (Fig. 4B). In Fig. 4C, we present the % changes for regions R1, 2 and 3, under P deficient conditions, showing that the % of roots distributed between these 3 regions were similar in nature between SC103 and BTx635.

### P acquisition versus utilization

Under sufficient Pi conditions (200 µM), SC103 exhibited a significant enhancement in P uptake (72% more shoot Pi content) than BTx635. As expected, Pi stress reduced uptake in both cultivars; however, P uptake was still significantly higher in SC103. Here, SC103 accumulated 50%, 64% and 43% more P in their shoots, compared with BTx635, under the three imposed Pi stress conditions, respectively (Table 1). In both cultivars, the reduction in P shoot content displayed a similar pattern. Interestingly, although we observed significant differences in P acquisition, between these two cultivars, no physiologically significant differences were observed in shoot and root P concentration under sufficient and deficient Pi conditions (Table 1). In addition, we observed higher P concentrations (*P* < 0.001) and P content (*P* < 0.001) in SC103 seeds, compared with BTx635 (Supplementary Fig. 1). This observed increase in plant P content, in SC103, is a reflection of its larger root system. Based on these findings, the lack of a difference in shoot and root P concentration between both cultivars, and the much larger P content in SC103, is consistent with P acquisition being a major contributor to enhanced P efficiency in SC103.

**Table 1:** Shoot and root phosphorous (P) concentration and content in sorghum lines SC103 and BTx635 grown under increasing phosphate (Pi) treatments

Treatment	Shoot P content (µg)		Shoot P concentration (mg g^−1^)		Root P content (µg)		Root P concentration (mg g^−1^)	
	SC103	BTx635	SC103	BTx635	SC103	BTx635	SC103	BTx635
0 μM	71 ± 3 c***	50 ± 3 c	1.4 ± 0.1 b	1.4 ± 0.03 c	60 ± 2 c***	32 ± 2 c	1.7 ± 0.1 c	1.4 ± 0.1 c
2.5 μM	79 ± 5 bc**	48 ± 5 c	1.5 ± 0.1 b	1.3 ± 0.1 c	70 ± 4 c***	34 ± 2 c	1.7 ± 0.04 c**	1.4 ± 0.1 c
10 μM	187 ± 20 b*	124 ± 17 b	2.6 ± 0.3 b	2.6 ± 0.3 b	142 ± 22 b*	78 ± 9 b	3.2 ± 0.3 b	3.0 ± 0.3 b
200 μM	599 ± 53 a**	349 ± 17 a	6.5 ± 0.5 a	6.3 ± 0.2 a	280 ± 28 a***	121 ± 6 a	6.3 ± 0.3 a	5.7 ± 0.2 a

Plants were grown in a hydroponic pouch system, with the indicated four different Pi conditions, and were harvested 12 days after transplanting. Data shown are means ± SE (n = 6). Different lowercase letters indicate significant differences (*P* < 0.05) among the Pi conditions in the same genotype, as determined by Tukey’s HSD tests. Asterisks indicate significant differences between genotypes in the same condition, as determined by Student’s t test analysis: *P < 0.05, **P < 0.01, ***P < 0.001.

We have recently, from a genetic and physiological analysis of sorghum P efficiency, determined that when dissecting sorghum P use efficiency into P utilization efficiency (PUE) and P acquisition efficiency (PAE), PAE accounts for the majority of the genetic contribution to P efficiency. Specifically, we determined in our study that PAE contributes 82% to the overall PAE, whereas PUE accounts for 18% (Bernardino et al. 2019). A similar strong genetic influence of PAE on P efficiency was previously reported in maize by Parentoni et al. (2010), where PAE explained approximately 80% of total P efficiency.

### Pi stress impacts root anatomy in both SC103 and BTx635

**Fig. 5:**
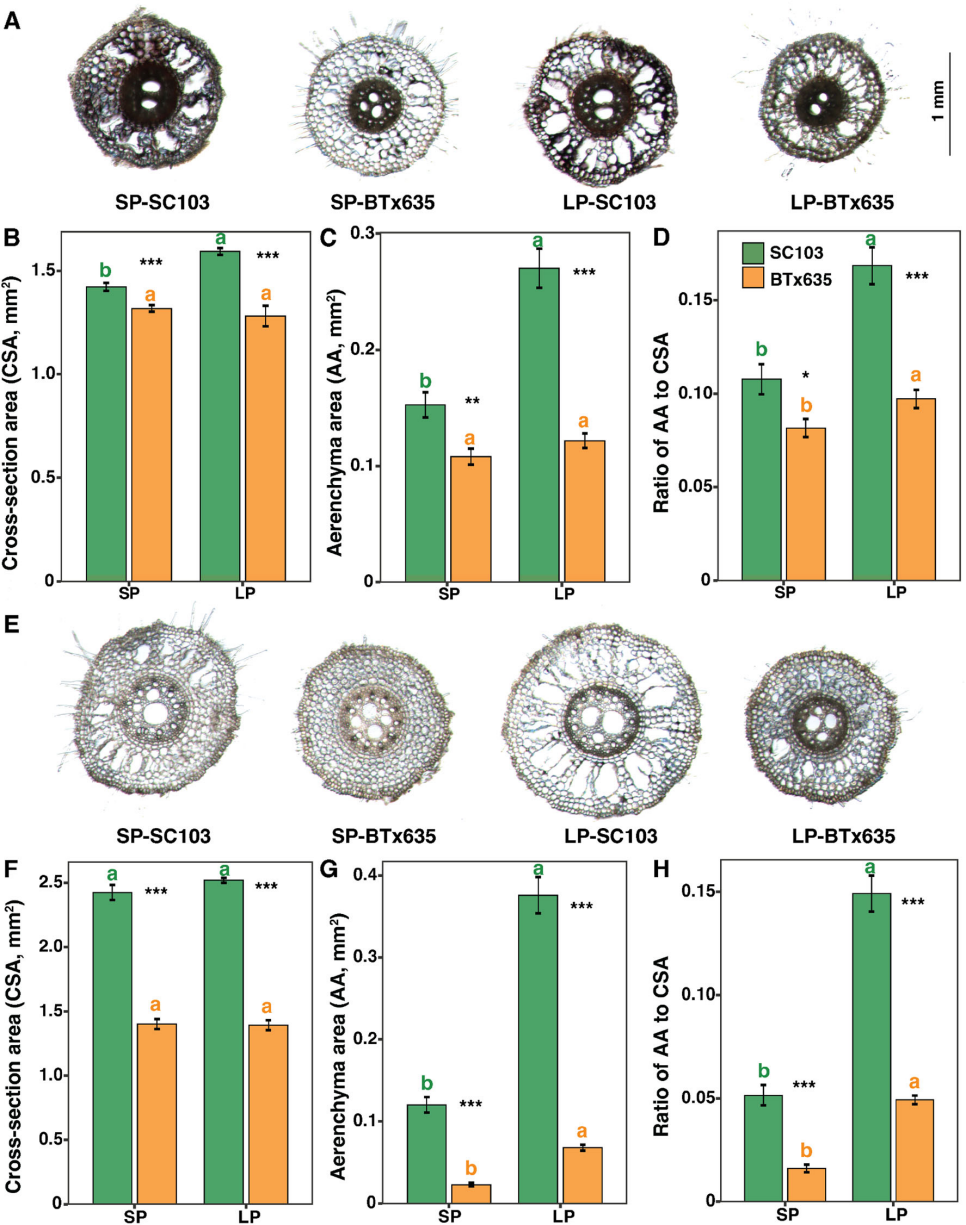
Primary root anatomy of sorghum cultivars SC103 and BTx635 grown for 10 days in hydroponics under sufficient Pi (SP, 200 μM) and low Pi (LP, 2.5 μM) conditions. Representative transverse images of primary root cross sections, collected 5 cm below the stem-root junction (**A**), and ~ 5 cm from the primary root tip (**E**) (see Fig. 4A). Histograms represent root cross-sectional area, total root cortical aerenchyma area (AA), and proportion of root cross section occupied by aerenchyma, near the stem root junction (**B**–**D**) and ~ 5 cm from the root tip (**F**–**H**) of the SC103 (green) and BTx635 (orange) primary root, respectively. Data shown are means ± SE (n = 6). Asterisks indicate significant differences between the cultivars, under the same condition, as determined by Student’s t test: for these assays, significance differences are indicated as follows: *P < 0.05; **P < 0.01; ***P < 0.001. Different lowercase letters indicate significant differences (*P* < 0.05) between the two Pi concentrations, in the same genotype, as determined by Tukey’s HSD tests. *CSA* cross section area (mm^2^), *AA* total root cortical aerenchyma area, (mm^2^). Scale bar applies to all images

**Fig. 6:**
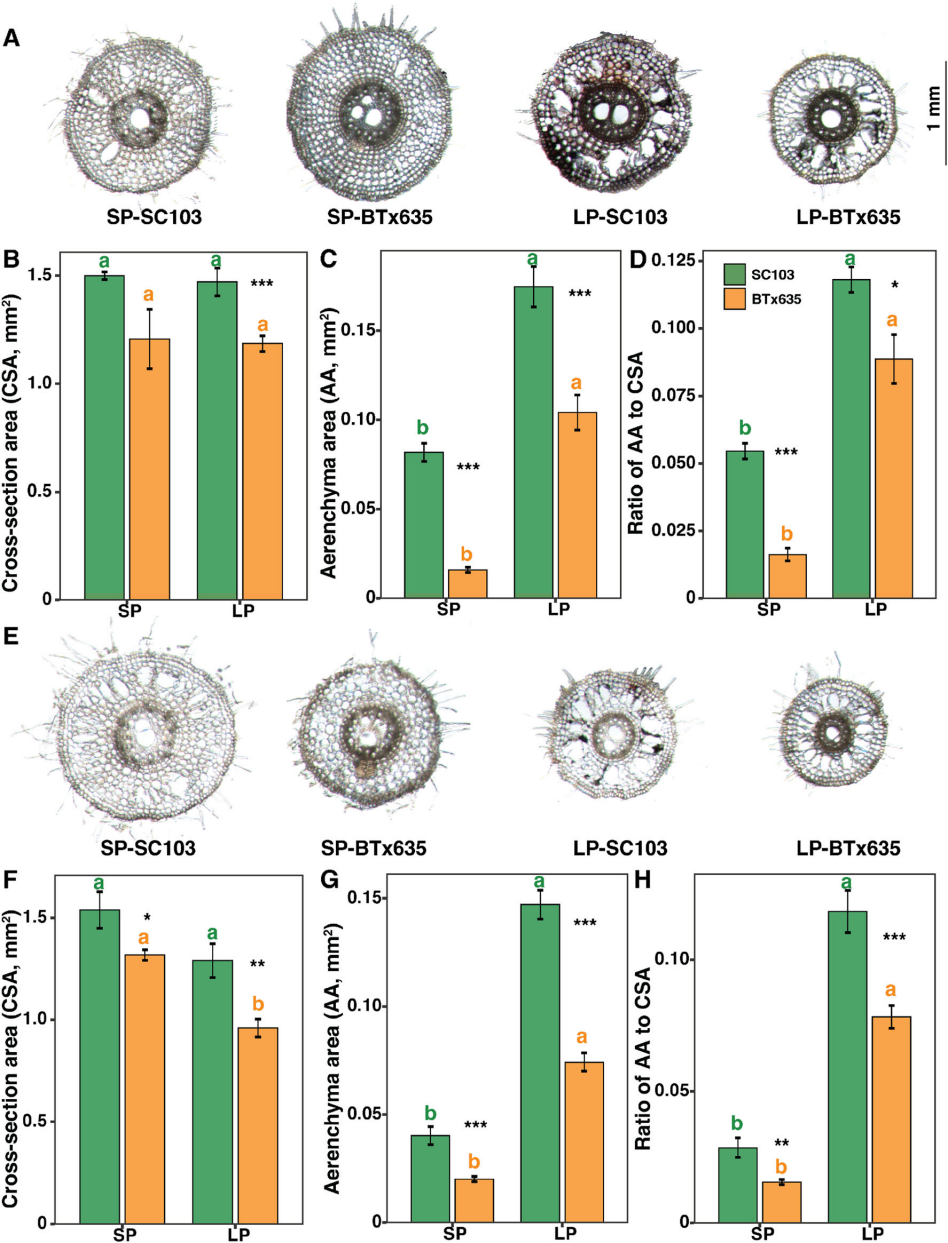
Lateral root anatomy of sorghum cultivars SC103 and BTx635 grown for 10 days in hydroponics under sufficient Pi (SP, 200 μM) and low Pi (LP, 2.5 μM) conditions. Representative transverse images of lateral root cross sections, collected 5 cm below the primary-lateral root junction (**A**), and ~ 5 cm from the lateral root tip (**E**) (see Fig. 4A). Histograms represent root cross-sectional area, total root cortical aerenchyma area (AA), and proportion of root cross section occupied by aerenchyma, near the primary-lateral root junction (**B**–**D**) and ~ 5 cm from the root tip (**F**–**H**) of the SC103 (green) and BTx635 (orange) lateral root, respectively. Data shown are means ± SE (n = 6). Asterisks indicate significant differences between the cultivars, under the same condition, as determined by Student’s t test: for these assays, significance differences are indicated as follows: *P < 0.05; **P < 0.01; ***P < 0.001. Different lowercase letters indicate significant differences (*P* < 0.05) between the two Pi concentrations, in the same genotype, as determined by Tukey’s HSD tests. *CSA* cross section area (mm^2^); *AA* total root cortical aerenchyma area (mm^2^). Scale bar applies to all images

Parameters associated with root anatomy, such as root cross-sectional area (CSA), total root cortical aerenchyma area (AA) and the proportion of root cross sectional area occupied by aerenchyma (AA/CSA), were evaluated (Figs. 5, 6, and Supplementary Fig. 2). Root segments were excised, from locations as indicated in Fig. 4A, and cross sections prepared for anatomical studies. Primary root cross sections, excised from near the stem-root junction and at least 5 cm back from the root tip, indicated the presence of extensive aerenchyma (Fig. 5A, E). However, under both sufficient Pi (200 μM; SP) and low Pi (2.5 μM; LP) conditions, in SC103, the AA was significantly larger, as was the AA/CSA ratio, compared with BTx635 (Fig. 5B–D, F–H).

A similar pattern was also observed for lateral root anatomy, near both the stem-root junction and at least 5 cm back from the lateral root tip. Again, SC103 had the larger AA and AA/CSA ratio, compared with BTx635, with these differences being enhanced under LP conditions (Fig. 6A–H). Crown root anatomy was also investigated, from the same locations as described above, and again SC103 had the larger AA and AA/CSA ratio, compared with BTx635, with these differences also being enhanced under LP conditions (Supplementary Fig. 2). These findings indicate that both sorghum lines develop aerenchyma under SP and LP conditions. Furthermore, aerenchyma development in SC103 was significantly greater compared with BTx635, under both SP and LP conditions.

### RSA response to N deficiency and drought stress

**Fig. 7:**
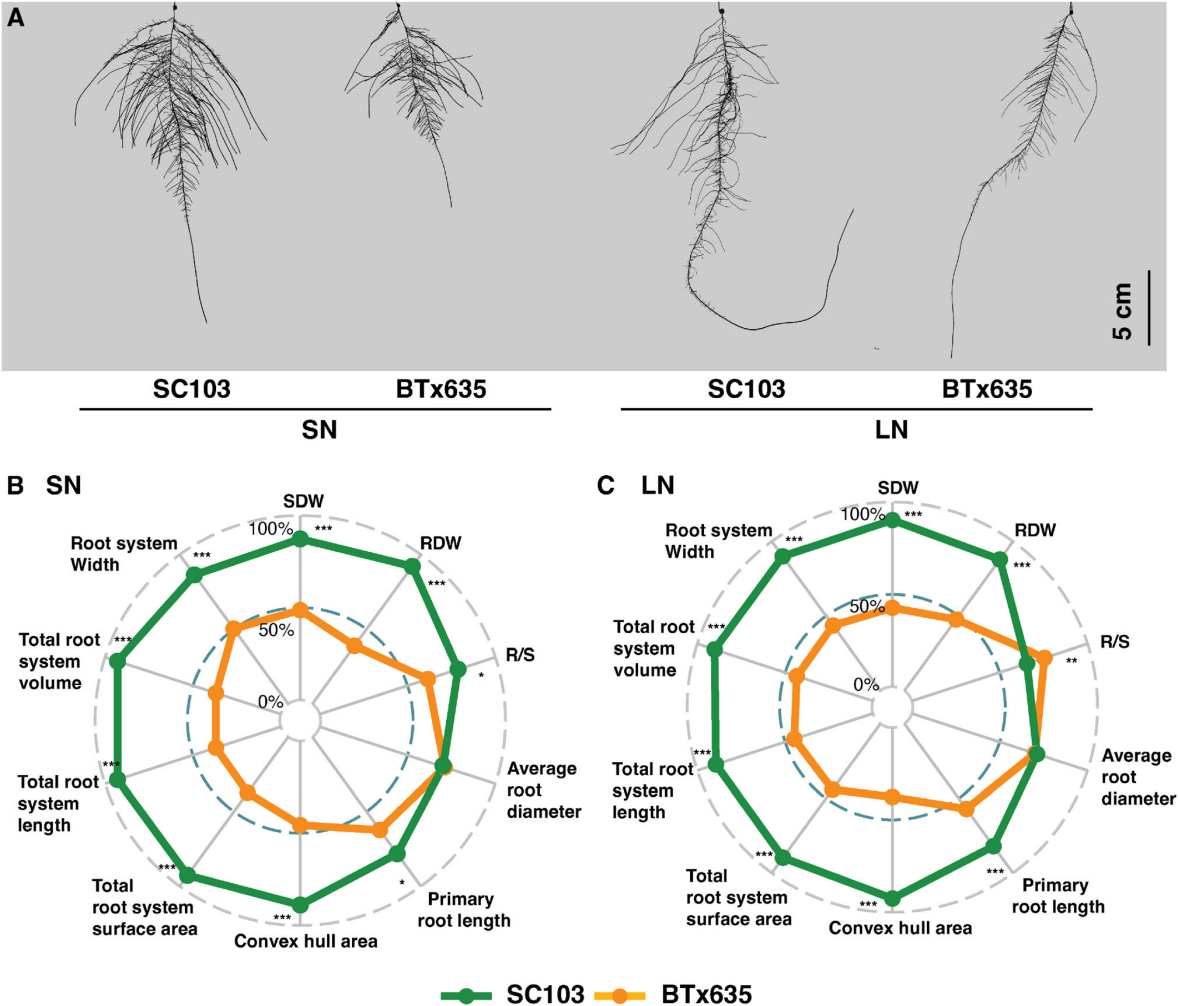
Root system architecture of sorghum cultivars, SC103 and BTx635, grown under sufficient N (SN, 4000 µM) and low N (LN, 400 µM) stress conditions. Plants were grown in a hydroponic pouch system and harvested at 10 Dat. **A** Representative root images of SC103 and BTx635, under SN and LN conditions. **B** Radar charts comparing the RSA traits of SC103 (green) and BTx635 (orange) under SN conditions. **C** Radar charts comparing the root system architecture traits of SC103 (green) and BTx635 (orange) under LN conditions. Asterisks indicate significant differences between the cultivars, under the same condition, as determined by Student’s t test: for these assays, significance differences are indicated as follows: *P < 0.05; **P < 0.01; ***P < 0.001. *SDW* shoot dry weight, *RDW* root dry weight, *R/S* root:shoot ratio

Parameters associated with biomass and RSA were assessed in response to an imposed N stress. Here, SC103 exhibited a larger root system, compared with BTx635, under both sufficient N (SN) and low N (LN) conditions (Fig. 7A and Supplementary Table 2). As shown in radar plots presented in Fig. 7B, under SN, all root and biomass traits, with the exception of average root diameter, were significantly larger in SC103 compared with BTx635. A similar pattern was observed under LN, except that, again, average root diameter was equivalent between the two cultivars, and BTx635 established a higher R/S ratio, compared to SC103 (Fig. 7C and Supplementary Table 2), due largely to its reduction in shoot biomass. These findings support the notion that the SC103 genetic composition can confer superior performance, relative to BTx635, under both P and N stress conditions.

**Fig. 8:**
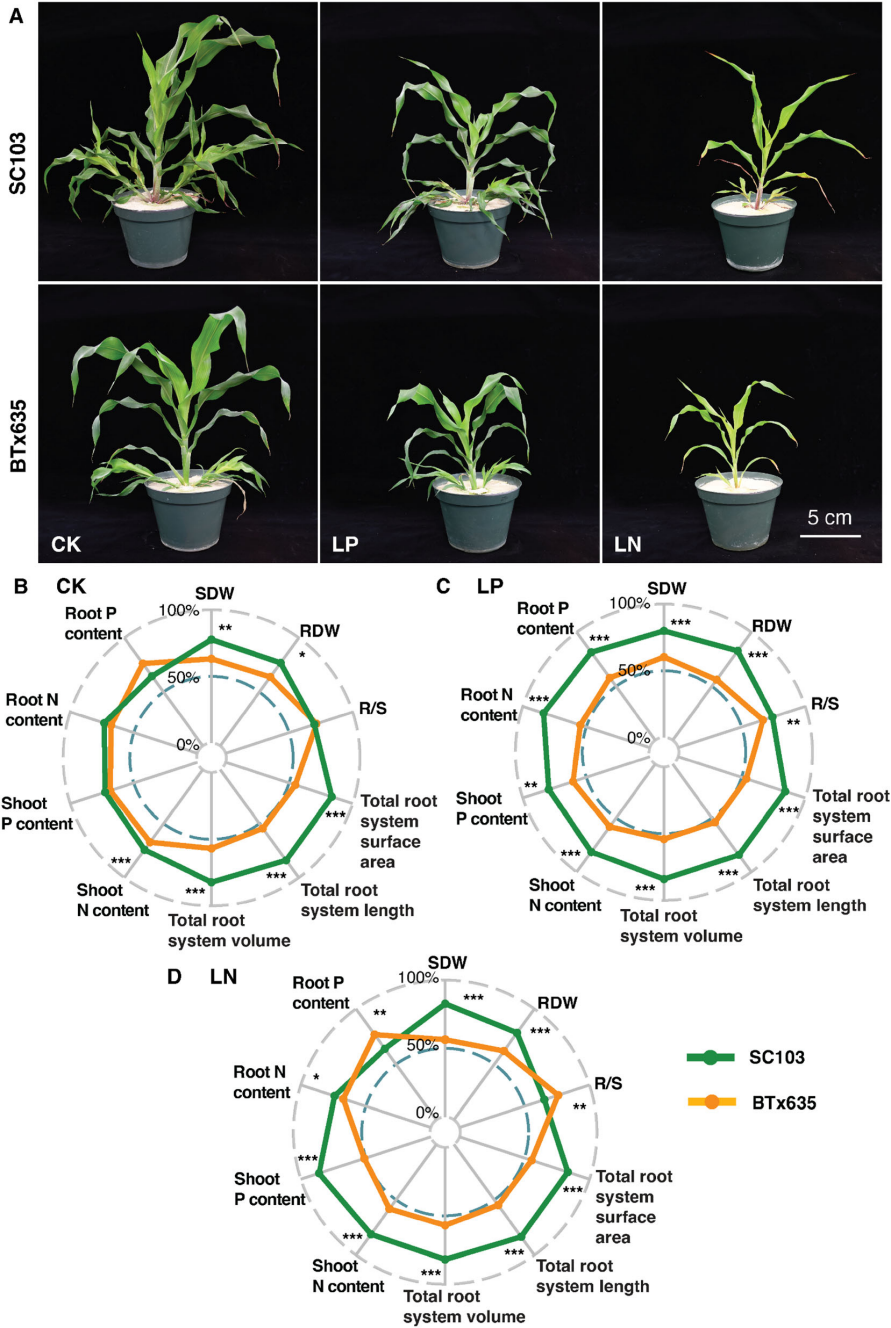
Phenotypic differences for sorghum cultivars SC103 and BTx635 grown under control (CK), low Pi (LP, 75 μM), and low N (LN, 600 µM) stress conditions. **A** Representative images of SC103 and BTx635, under CK, LP and LN stress conditions. Images were taken 28 Dat. **B** Radar plots quantifying the RSA traits of SC103 and BTx635, under CK conditions. **C** Radar plots quantifying the RSA traits of SC103 and BTx635, under LP conditions. **D** Radar plots quantifying the RSA traits of SC103 and BTx635, under LN conditions. Asterisks indicate significant differences between the cultivars under CK, LP and LN conditions, as determined by Student’s t test: for these assays, significance differences are indicated as follows: *P < 0.05; **P < 0.01; ***P < 0.001. *SDW* shoot dry weight, *RDW* root dry weight, *R/S* root:shoot ratio

To explore the effect of plant development, on shoot and RSA traits, in these two sorghum cultivars, experiments were next conducted by growing plants in silica sand, as substrate. Here, we tested the effects of LP and LN on RSA and growth. As anticipated, shoot biomass was reduced under both LP and LN conditions (Fig. 8A). Under control conditions, the relative changes in RSA traits and growth characteristics, between SC103 and BTx635, are shown in Fig. 8B (see also Supplementary Fig. 3 and Supplementary Table 3). The noteworthy differences were in total root system volume, length, and surface area, which were always higher in SC103. Under LP growth conditions, significant differences were observed for all characteristics examined, with SC103 having the superior traits (Fig. 8C; see also Supplementary Fig. 3 and Supplementary Table 3). Under LN growth conditions, significant differences were also observed for all characteristics examined, with SC103 having the superior traits, with the exception that BTx635 had a higher root Pi content and R/S ratio (Fig. 8D; see also Supplementary Fig. 3 and Supplementary Table 3). These findings are equivalent to those observed for plants grown in the LP hydroponic pouch systems.

**Fig. 9:**
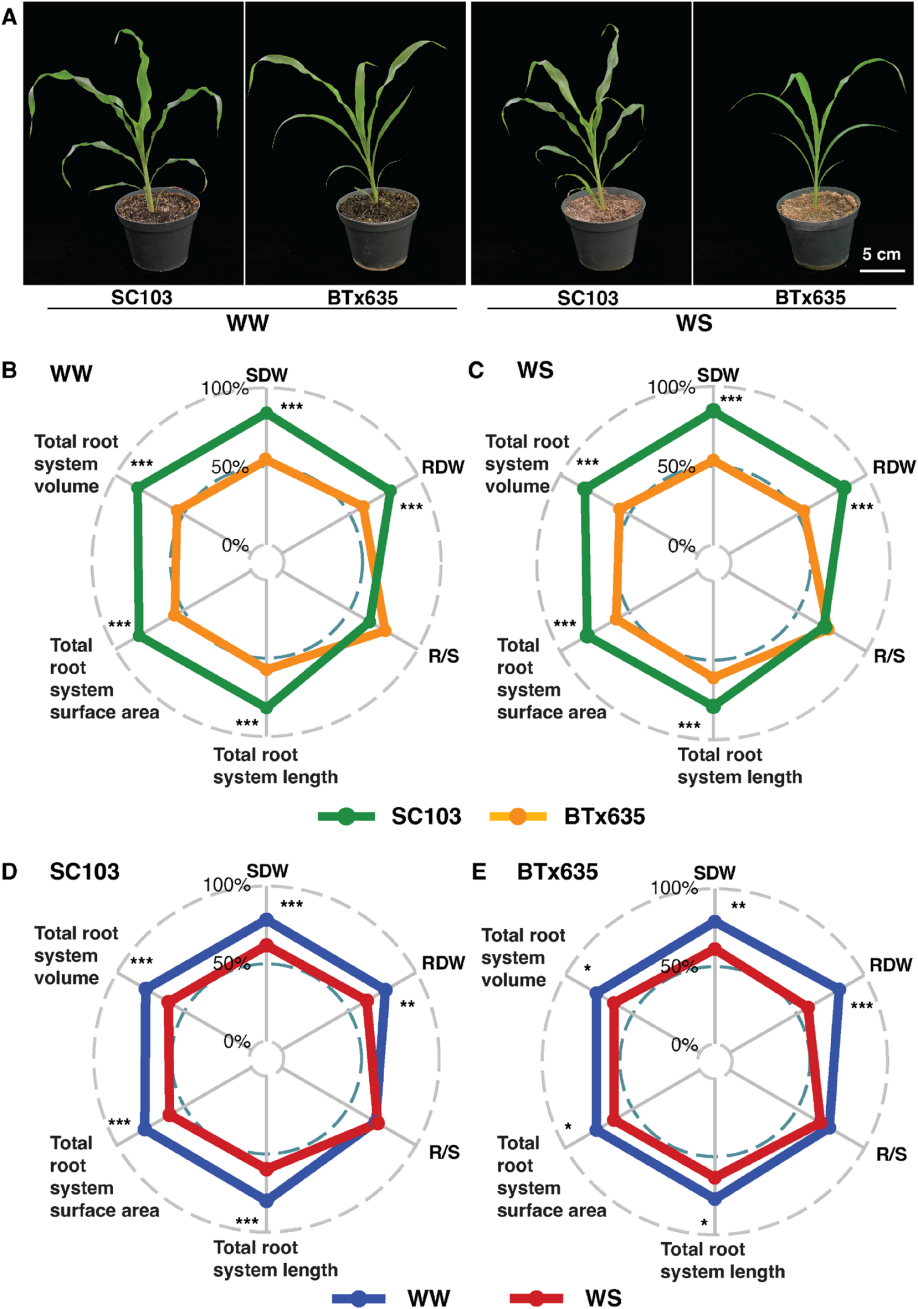
Phenotypic differences for sorghum cultivars SC103 and BTx635 in response to water stress. Plants were grown in potting mix for 28 Dat. At the 14-day timepoint, plants were separated into two groups; a control group was well-watered (WW), whereas water was withheld from the water stress (WS) group. **A** Representative images of SC103 and BTx635, grown under WW and WS conditions. **B** Radar plots quantifying the RSA traits of SC103 and BTx635, under WW treatment. **C** Radar plots quantifying the RSA traits of SC103 and BTx635, under WS treatment. **D** Radar plots quantifying the RSA traits of SC103 under WW and WS treatment. **E** Radar plots quantifying the RSA traits of BTx635 under WW and WS treatment. Asterisks indicate the significant differences between the cultivars and treatments, as determined by Student’s t test: for these assays, significance differences are indicated as follows: *P < 0.05; **P < 0.01; ***P < 0.001. *SDW* shoot dry weight, *RDW* root dry weight, *R/S* root:shoot ratio

Drought conditions can impair crop performance and yield. To explore whether SC103 and BTx635 are resilient to an imposed water stress condition, plants were grown in potting mix for four weeks. At the two week timepoint, the plants were divided into two equal groups; the control group continued to be well-watered (WW), and the test group was subjected to water stress (WS), by withholding water (Fig. 9A). As might be anticipated, SC103 displayed superior growth and RSA characteristics, compared with BTx635, under both WW and WS treatments, except for R/S ratio (Fig. 9B, C; see also Supplementary Table 4). The impact of WS on growth and RSA traits for each sorghum line is shown in Fig. 9D, E (see also Supplementary Table 4), and indicates that, for both lines, all parameters were reduced under WS conditions.

## Discussion

The root system is of fundamental importance for seedling establishment, to enable plant growth and survival through its pivotal role in uptake of essential nutrients and water. During seedling establishment, the majority of plant development occurs within the root system. The early establishment of roots has been closely linked to grain yield, rather than to the subsequent stages of root development (Cai et al. 2012). Furthermore, overexpression of *OsMYB4P*, an R2R3-type MYB transcriptional activator, in rice seedlings increased seminal and lateral root length and density, which enhanced phosphate acquisition (Yang et al. 2014). With regards to breeding for crop improvement, attention to identifying traits, associated with early establishment of an extensive root system, would likely provide pathways for developing lines with efficient growth and subsequent yield enhancement.

### RSAs for efficient performance under limiting Pi and/or N conditions

Our findings establish that, under phosphate stress conditions, neither SC103 nor BTx635 developed RSA traits equivalent to those anticipated for plants that are optimized for yield performance on soils in which available Pi is located primarily within the topsoil (Liang et al. 2010; Lynch 2011). Although our conditions used for plant growth and root trait analysis utilized hydroponic pouches, sand culture, and potting soil, the RSAs obtained under SP and LP conditions provide insight into the genetic program(s) underlying root development and growth. For example, in Fig. 2, under SP, the root systems developed by both SC103 and BTx635 occupied a remarkably small volume; this pattern would be equivalent to root growth in the topsoil (see Fig. 1). If these two test cultivars were genetically adapted, via breeding programs, to generate a shallow root system, then we would anticipate that exposure to LP conditions would produce more and longer horizontally-oriented roots. As shown in Fig. 2, an entirely different response was observed, in which the root systems expanded both laterally and vertically to occupy a greatly enlarged volume (Figs. 3, 4). In this regard, both the primary and lateral roots responded in a reactive manner to a reduction in available Pi.

The growth systems employed in our study did not mimic the soil conditions under which the differences in yield performance, between SC103 and BTx635, were recorded. It is noteworthy, that these plants were grown under field conditions, where available Pi, at limiting levels, was confined to the topsoil. However, this stratification of soil P concentration to higher levels in the topsoil, in the field, does not apply to our studies on LN or water availability, as these resources are mobile and are generally distributed deeper within the soil profile. Again, the root systems that developed under LN (Figs. 7, 8) and WS (Fig. 9), did not conform to the predicted topsoil model. Rather, under LN and WS, the root patterns for both cultivars, were similar to those observed under LP. One crucial objective in the future will be to conduct field validation to ground truth the differences in N efficiency and performance under drought conditions for both sorghum cultivars. It should be noted, as mentioned in the “Materials and methods”, that the differences in P efficiency for SC103 and BTx635 were first determined and quantified in the field by measuring grain yield on a low P field site.

The findings from the current study provide insight into an efficient pathway for developing crops with enhanced soil performance characteristics. In general, as with SC103 and BTx635, many crop plants utilize an RSA equivalent to an inverted triangular root system (Fig. 1B). Hence, this RSA removes the need to engineer dramatic genetic reprogramming, in order to convert from an inverted root system into one in which the majority of the roots are constrained to a more horizontal plane. In this regard, SC103 may well provide a valuable resource for such breeding activities. First, it has genetic characteristics associated with enhanced resource allocation, resulting in more extensive aerenchyma development (Figs. 5, 6, and Supplemental Fig. 2), which optimizes the cost associated with the generation and maintenance of a large root system (Lynch 2018). The resultant enhanced root system surface area increased the capacity for resource acquisition, which in this study resulted in enhanced P, N and water uptake.

### Aerenchyma as a key growth and yield determinant

The amount of root cortical aerenchyma can vary, depending on plant species, genotype, developmental stage, root class, and position along the root axis, or within the transverse section (Armstrong 1972; Bouranis et al. 2006; Evans 2004; Kawai et al. 1998). Additionally, both nutrient limitation and WS can induce aerenchyma development (Chimungu et al. 2015; Postma and Lynch 2011; Saengwilai et al. 2014). The basis for the observed difference in root system size, between SC103 and BTx635, may reflect a significant difference in the degree to which aerenchyma developed in these two lines. For both primary and lateral roots, in the recently developed zone of these root types, significant differences in aerenchyma existed between SC103 and BTx635, under both SP and LP conditions; here, the extent of aerenchyma development was greatly enhanced under SP and LP conditions in SC103 (Figs. 5, 6). Given that aerenchyma develops in these root systems, under both SP and LP conditions, this may reflect the operation of a constitutive regulatory genetic program.

As SC103 establishes an enhanced level of aerenchyma, relative to BTx635, this must reflect the presence of different genetic elements in these two cultivars. This difference likely indicates alterations in gene promoter properties in these two lines. For example, Schneider et al. (2023) recently reported that *ZmbHLH121* acts as a positive regulator of root cortical aerenchyma formation. Thus, differential expression of this gene homolog, in SC103 and BTx635, could contribute to their differences in aerenchyma. Therefore, root cortical aerenchyma represents a promising target for the breeding of crop cultivars with improved stress tolerance, resilience, and carbon sequestration. Additionally, based on the observed enhancement of aerenchyma, under LP conditions, these lines appear to possess both constitutive and inductive genetic components. A similar conclusion can be drawn for the operation of regulatory components controlling root growth traits under SP and LP conditions (Fig. 3).

### Breeding for superior RSAs

Based on its performance in our studies, SC103 represents an important genetic resource for identifying genes, or genomics regions, that can be employed for breeding crops with an RSA for fitness under nutrient and/or water limitations. Furthermore, the high yielding capacity of SC103, obtained on field soils with low available Pi, also presents opportunities for RSA traits, measured under laboratory and greenhouse conditions, to be integrated into breeding programs for nutrient utilization efficiencies, including N, P, K, and also water. Presently, considerable sorghum genetic resources are available, including different mapping populations, and multiple pangenomes. These provide an important platform for identifying genomic regions and/or genes underlying these traits, which can be used for crop improvement by employing genome-design-based breeding, marker-assisted introgression, and gene-based editing. It is noteworthy that the constitutive (hard-wired) versus induced genetic component of the SC103 RSA traits could facilitate trait engineering into target crops due to a reduced impact by genome × environment interactions. In this manner, crop improvement be would available across a wider range of soil types and agroecologies. Lastly, as SC103 is more efficient in terms of P and N acquisition and drought resilience, this could allow for the efficient breeding for all three traits from one genetic source.

## Conclusions

The grain yield differential, between SC103 and BTx635, established under low Pi field conditions, was clearly reflected in the measured RSA and growth characteristics, determined under LP, LN, and WS conditions (Figs. 2, 3, 4, 7, 8, 9). Given that SC103 and BTx635 have equivalently structured root systems (Fig. 2), these performance differences likely reflect either changes in the operational characteristics of a putative master controller, or higher expression levels of key genes involved in resource allocation. These differences resulted in a larger and more dynamic root system in SC103, thereby supporting an enhanced shoot system, resulting in the measured higher yield. Identifying the genetic determinants responsible for the superior performance of SC103 would provide a valuable resource for both further research into the establishment of superior RSA traits and to facilitate breeding programs.

### Supplementary Information

Below is the link to the electronic supplementary material.

Supplementary file1Supplementary Fig. 1 Analysis of seed P and N in SC103 and BTx635. (A) Seed P concentration, (B) seed N concentration, (C) seed P content, and (D) seed N content. Supplementary Fig. 2 Crown root anatomy of sorghum cultivars SC103 and BTx635 grown for 10 days in hydroponics under sufficient Pi (SP, 200 μM) and low Pi (LP, 2.5 μM) conditions. Representative transverse images of crown root cross sections, collected 5 cm below the stem-root junction (A), and ~5 cm from the crown root tip (E) (see Fig. 4A). Histograms represent root cross-sectional area, total root cortical aerenchyma area (AA), and proportion of root cross section occupied by aerenchyma, near the stem-root junction (B, C, D) and ~5 cm from the root tip (F, G, H) of the SC103 (green) and BTx635 (orange) crown root, respectively. Data shown are means ± SE (n=6). Asterisks indicate significant differences between the cultivars, under the same condition, as determined by Student’s t-test: For these assays, significance differences are indicated as follows: * P < 0.05; ** P < 0.01; *** P < 0.001. Different lowercase letters indicate significant differences (P < 0.05) between the two Pi concentrations, in the same genotype, as determined by Tukey’s HSD tests. CSA, cross section area (mm2); AA, total root cortical aerenchyma area (mm2). Scale bar applies to all images. Supplementary Fig. 3 SC103 and BTx635 RSA and growth data used to develop the radar plots in Fig. 8 B-D. Histograms represent shoot dry weight (A), root dry weight (B), root:shoot ratio (C), total root system length (D), total root system surface area (E), root system volume (F), shoot N content (G), shoot P content (H), root N content (I), root P content (J). Data were collected from plants grown in pots with silica sand as substrate under control (CK), low Pi (LP, 75 μM), or low N (LN, 600 μM). Data shown are means ± SE (n=6). Asterisks indicate significant differences between the cultivars, under the same condition, as determined by Student’s t-test: For these assays, significance differences are indicated as follows: * P < 0.05; ** P < 0.01; *** P < 0.001. Different lowercase letters indicate significant differences (P < 0.05) between the two Pi concentrations, in the same genotype, as determined by Tukey’s HSD tests (PPTX 17516 KB)

Supplementary file2 (DOCX 25 KB)

## Data Availability

All data generated or analyzed during this study are available from the corresponding author upon request.
