# Supplementary figures and images for: Constitutive basis of root system architecture: uncovering a promising trait for breeding nutrient- and drought-resilient crops
Source: aBIOTECH. 2023 Sep 15;4(4):315–31. doi: 10.1007/s42994-023-00112-w (PMC10721591; doi:10.1007/s42994-023-00112-w)

## Slide 1
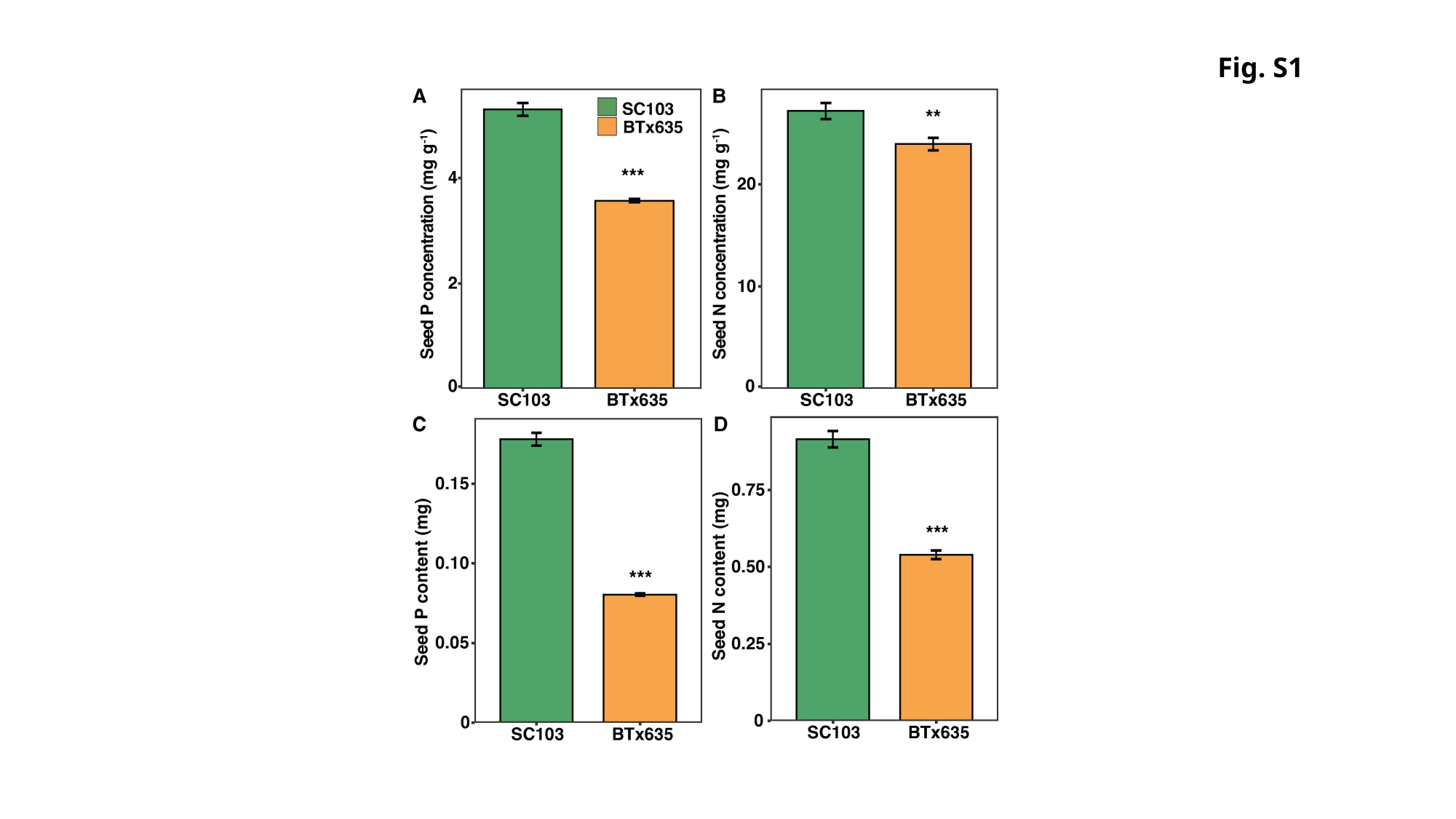

Fig. S1

## Slide 2
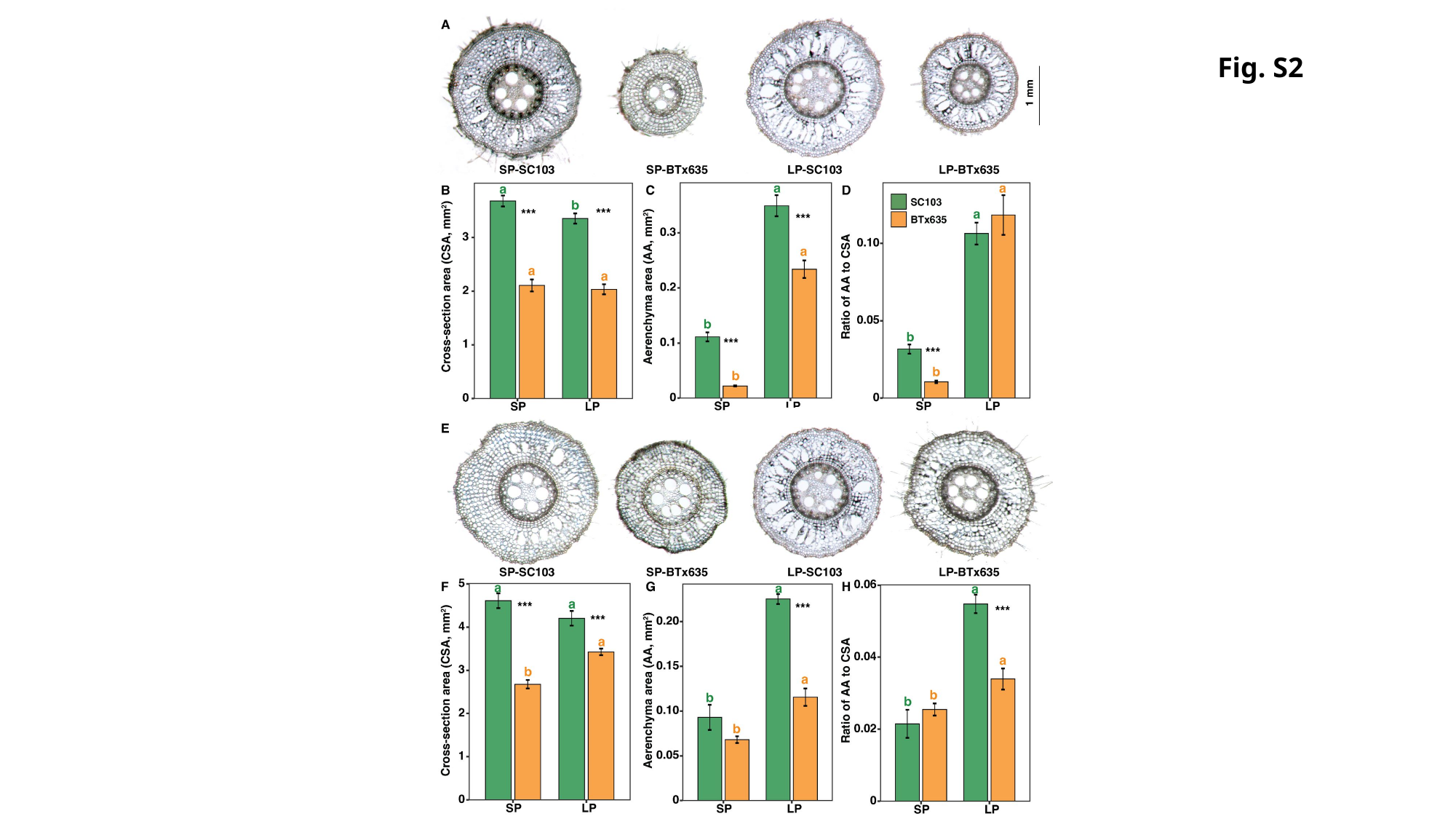

Fig. S2

## Slide 3
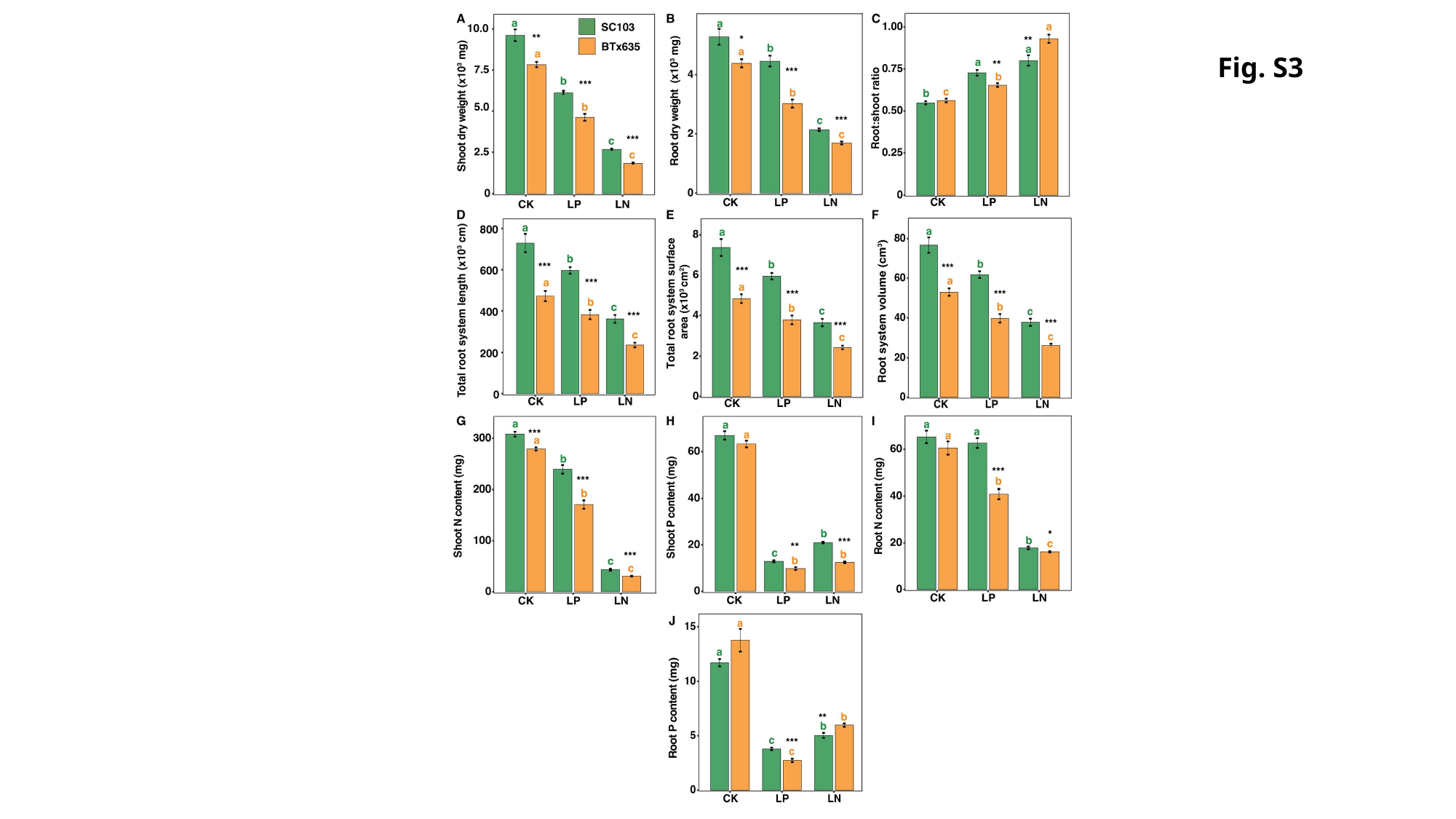

Fig. S3

Supplement: Supplementary file 1 — Supplementary file1Supplementary Fig. 1 Analysis of seed P and N in SC103 and BTx635. (A) Seed P concentration, (B) seed N concentration, (C) seed P content, and (D) seed N content. Supplementary Fig. 2 Crown root anatomy of sorghum cultivars SC103 and BTx635 grown for 10 days in hydroponics under sufficient Pi (SP, 200 μM) and low Pi (LP, 2.5 μM) conditions. Representative transverse images of crown root cross sections, collected 5 cm below the stem-root junction (A), and ~5 cm from the crown root tip (E) (see Fig. 4A). Histograms represent root cross-sectional area, total root cortical aerenchyma area (AA), and proportion of root cross section occupied by aerenchyma, near the stem-root junction (B, C, D) and ~5 cm from the root tip (F, G, H) of the SC103 (green) and BTx635 (orange) crown root, respectively. Data shown are means ± SE (n=6). Asterisks indicate significant differences between the cultivars, under the same condition, as determined by Student’s t-test: For these assays, significance differences are indicated as follows: * P < 0.05; ** P < 0.01; *** P < 0.001. Different lowercase letters indicate significant differences (P < 0.05) between the two Pi concentrations, in the same genotype, as determined by Tukey’s HSD tests. CSA, cross section area (mm2); AA, total root cortical aerenchyma area (mm2). Scale bar applies to all images. Supplementary Fig. 3 SC103 and BTx635 RSA and growth data used to develop the radar plots in Fig. 8 B-D. Histograms represent shoot dry weight (A), root dry weight (B), root:shoot ratio (C), total root system length (D), total root system surface area (E), root system volume (F), shoot N content (G), shoot P content (H), root N content (I), root P content (J). Data were collected from plants grown in pots with silica sand as substrate under control (CK), low Pi (LP, 75 μM), or low N (LN, 600 μM). Data shown are means ± SE (n=6). Asterisks indicate significant differences between the cultivars, under the same condition, as dete [file 42994_2023_112_MOESM1_ESM.pptx]
