# Supplementary material for: Constitutive basis of root system architecture: uncovering a promising trait for breeding nutrient- and drought-resilient crops
Source: aBIOTECH. 2023 Sep 15;4(4):315–31. doi: 10.1007/s42994-023-00112-w (PMC10721591; doi:10.1007/s42994-023-00112-w)
Supplement: Supplementary file 2 — Supplementary file2 (DOCX 25 KB) [file 42994_2023_112_MOESM2_ESM.docx]

**Supplementary Table 1** Root system architecture of sorghum cultivars SC103 and BTx635 grown under control and the indicated phosphate (Pi) stress treatment.

| Time | Treatment | Average root diameter (mm) | | Total root system volume (cm^3^) | | Convex hull area (cm^2^) | | Root dry weight (mg) | |
| --- | --- | --- | --- | --- | --- | --- | --- | --- | --- |
|  |  | SC103 | BTx635 | SC103 | BTx635 | SC103 | BTx635 | SC103 | BTx635 |
| 7 Dat | 0 μM | 0.348 ± 0.002 b | 0.353 ± 0.005 a | 0.25 ± 0.02 a ** | 0.16 ± 0.01 ab | 164 ± 17 a * | 119 ± 11 a | ^a^NA | NA |
|  | 2.5 μM | 0.348 ± 0.003 b | 0.351 ± 0.004 a | 0.27 ± 0.02 a ** | 0.17 ± 0.01 a | 149 ± 15 ab * | 112 ± 6 a | NA | NA |
|  | 10 μM | 0.364 ± 0.005 ab | 0.350 ± 0.007 a | 0.26 ± 0.02 a ** | 0.18 ± 0.01 a | 111 ± 11 b | 97 ± 10 a | NA | NA |
|  | 200 μM | 0.372 ± 0.008 a | 0.370 ± 0.009 a | 0.14 ± 0.01 b | 0.12 ± 0.01 b | 58 ± 6 c * | 44 ± 2 b | NA | NA |
| 9 Dat | 0 μM | 0.336 ± 0.002 a * | 0.345 ± 0.004 a | 0.34 ± 0.02 ab *** | 0.20 ± 0.02 ab | 221 ± 23 a | 165 ± 17 a | NA | NA |
|  | 2.5 μM | 0.338 ± 0.002 a | 0.342 ± 0.003 a | 0.36 ± 0.03 a ** | 0.23 ± 0.01 a | 181 ± 16 a | 167 ± 11 a | NA | NA |
|  | 10 μM | 0.342 ± 0.003 a | 0.342 ± 0.004 a | 0.37 ± 0.04 a ** | 0.23 ± 0.01 a | 155 ± 18 a | 123 ± 8 a | NA | NA |
|  | 200 μM | 0.348 ± 0.007 a | 0.349 ± 0.005 a | 0.24 ± 0.01 b *** | 0.16 ± 0.01 b | 71 ± 7 b | 60 ± 6 b | NA | NA |
| 12 Dat | 0 μM | 0.322 ± 0.003 b ** | 0.342 ± 0.002 ab | 0.46 ± 0.03 a *** | 0.30 ± 0.03 ab | 338 ± 39 a | 283 ± 31 a | 36 ± 2 a *** | 22 ± 2 a |
|  | 2.5 μM | 0.331 ± 0.005 b | 0.332 ± 0.001 ab | 0.51 ± 0.05 a * | 0.36 ± 0.03 a | 296 ± 27 a | 277 ± 14 a | 43 ± 3 a *** | 25 ± 1 a |
|  | 10 μM | 0.328 ± 0.005 b | 0.332 ± 0.006 b | 0.58 ± 0.07 a * | 0.36 ± 0.04 a | 243 ± 24 a * | 162 ± 11 b | 44 ± 3 a *** | 26 ± 1 a |
|  | 200 μM | 0.362 ± 0.006 a | 0.347 ± 0.009 a | 0.54 ± 0.07 a *** | 0.21 ± 0.01 b | 109 ± 6 b ** | 68 ± 6 c | 44 ± 4 a *** | 21 ± 1 a |

^a^ Data not available (NA)

Plants were grown in a hydroponic pouch system, under the indicated Pi conditions, and were harvested 12 Dat. Root system architecture was investigated at 7, 9 and 12 Dat. Data shown are means ± SE (n=6). Data shown are means ± SE (n=6). Asterisks indicate significant differences between the cultivars, under the same condition, as determined by Student’s t-test: For these assays, significance differences are indicated as follows: * *P* < 0.05; ** *P* < 0.01; *** *P* < 0.001. Different lowercase letters indicate significant differences (*P* < 0.05) under control and the indicated Pi conditions, in the same genotype, as determined by Tukey’s HSD tests.

**Supplementary Table 2** Root system architecture of sorghum cultivars SC103 and BTx635 grown under sufficient (SN) and low nitrogen (LN) treatments

| Treatment | Shoot dry weight (mg) | | Root dry weight (mg) | | Root :shoot ratio | | Average root diameter (mm) | | Primary root length (cm) | | Convex hull area (cm2) | | Root system surface area (cm2) | | Total root system length (cm) | | Total root system volume (cm3) | | Root system width (cm) | |
| --- | --- | --- | --- | --- | --- | --- | --- | --- | --- | --- | --- | --- | --- | --- | --- | --- | --- | --- | --- | --- |
|  | SC103 | BTx635 | SC103 | BTx635 | SC103 | BTx635 | SC103 | BTx635 | SC103 | BTx635 | SC103 | BTx635 | SC103 | BTx635 | SC103 | BTx635 | SC103 | BTx635 | SC103 | BTx635 |
| SN | 85.8±5 a*** | 47.8±4.6 a | 67.3±3.7 a*** | 28.4±1.2 a | 0.8±0.04 b* | 0.62±0.04 b | 0.34±0.004 | 0.35±0.002 | 26.6±1.9 b* | 21.2±1.4 b | 293±20 b*** | 150±11 a | 104±6 a*** | 42±2 a | 975±66 a*** | 387±21 a | 1.04±0.06 a*** | 0.41±0.02 a | 19.2±0.7 a*** | 11.2±0.8 a |
| LN | 32±0.5 b*** | 15.1±0.5 b | 35.8±1.2 b*** | 19.4±0.5 b | 1.12±0.03 a** | 1.29±0.03 a | 0.35±0.002 | 0.34±0.004 | 51.2±0.8 a*** | 35.6±1.4 a | 462±21 a*** | 188±16 a | 57±3 b*** | 28±2 b | 519±26 b*** | 260±17 b | 0.56±0.02 b*** | 0.27±0.01 b | 17.4±1.2 a*** | 8.4±0.7 b |

Plants were grown in a hydroponic pouch system and harvested at 10 Dat. Root system architecture was investigated at 10 Dat. Data shown are means ± SE (n=6). Asterisks indicate significant differences between the cultivars, under the same condition, as determined by Student’s t-test. For these assays, significance differences are indicated as follows: * *P* < 0.05; ** *P* < 0.01; *** *P* < 0.001. Different lowercase letters indicate significant differences (*P* < 0.05) between sufficient N (SN) and low N (LN) treatments, in the same genotype, as determined by Tukey’s HSD tests.

**Supplementary Table 3** Root system architecture of sorghum cultivars SC103 and BTx635 grown under control (CK), low nitrogen (LN) and low phosphate (LP) treatments

| Treatment | Shoot dry weight (mg) | | Root dry weight (mg) | | Root:shoot ratio | | Total root system surface area (cm^2^) | | Total root system length (cm) | | Total root system volume (cm3) | | Shoot N content (mg) | | Shoot Pi content (mg) | | Root N content (mg) | | Root Pi content (mg) | |
| --- | --- | --- | --- | --- | --- | --- | --- | --- | --- | --- | --- | --- | --- | --- | --- | --- | --- | --- | --- | --- |
|  | SC103 | BTx635 | SC103 | BTx635 | SC103 | BTx635 | SC103 | BTx635 | SC103 | BTx635 | SC103 | BTx635 | SC103 | BTx635 | SC103 | BTx635 | SC103 | BTx635 | SC103 | BTx635 |
| CK | 9.6 ± 0.4 a** | 7.8 ± 0.2 a | 5.3 ± 0.3 a* | 4.4 ± 0.1 a | 0.55 ± 0.01 b | 0.56 ± 0.01 c | 7367 ± 421 a*** | 4832 ± 214 a | 72908 ± 4502 a*** | 47184 ± 2476 a | 77 ± 4 a*** | 53 ± 2 a | 308 ± 5 a*** | 279 ± 3 a | 67 ± 1.8 a | 63 ± 1.4 a | 65 ± 2.6 a | 61 ± 2.8 a | 11.7 ± 0.3 a | 13.8 ± 1 a |
| LP | 6.1 ± 0.1 b** | 4.6 ± 0.2 b | 4.5 ± 0.2 b*** | 3 ± 0.1 b | 0.73 ± 0.02 a** | 0.65 ± 0.01 b | 5947 ± 161 b*** | 3783 ± 216 b | 59638 ± 1613 b*** | 38204 ± 2274 b | 62 ± 2 b*** | 40 ± 2 b | 239 ± 8 b*** | 171 ± 8 b | 13 ± 0.4 c** | 10 ± 0.6 b | 63 ± 2.1 a *** | 41 ± 2.1 b | 3.8 ± 0.1 c*** | 2.7 ± 0.2 c |
| LN | 2.7 ± 0.1 c** | 1.8 ± 0.1 c | 2.1 ± 0.04 c*** | 1.7 ± 0.05 c | 0.8 ± 0.03 a** | 0.93 ± 0.02 a | 3644 ± 186 c*** | 2428 ± 91 c | 36094 ± 1895 c*** | 23652 ± 1112 c | 38 ± 2 c*** | 26 ± 1 c | 43 ± 2 c*** | 31 ± 1 c | 21 ± 0.4 b*** | 13 ± 0.4 b | 18 ± 0.6 b * | 16 ± 0.3 c | 5 ± 0.2 b** | 6 ± 0.2 b |

Plants were grown in pots with silica sand substrate for 28 Dat under control (CK), low Pi (LP, 75 μM), and N stress (LN, 600 µM) conditions. Data shown are means ± SE (n=6). Asterisks indicate significant differences between the cultivars under CK, LP and LN conditions, as determined by Student’s t-test. For these assays, significance differences are indicated as follows: * *P* < 0.05; ** *P* < 0.01; *** *P* < 0.001. Different lowercase letters indicate significant differences (*P* < 0.05) among control (CK), low Pi (LP) and low N (LN) treatments, in the same genotype, as determined by Tukey’s HSD tests.

**Supplementary Table 4** Root system architecture of sorghum cultivars SC103 and BTx635 grown under well-watered (WW) and water stress (WS) treatments

| Treatment | Shoot dry weight (mg) | | Root dry weight (mg) | | Root:shoot ratio | | Total root system length (cm) | | Total root system surface area (cm^2^) | | Total root system volume (cm^3^) | |
| --- | --- | --- | --- | --- | --- | --- | --- | --- | --- | --- | --- | --- |
|  | SC103 | BTx635 | SC103 | BTx635 | SC103 | BTx635 | SC103 | BTx635 | SC103 | BTx635 | SC103 | BTx635 |
| WW | 2305±52 a*** | 1483±90 a | 327±6 a*** | 244±13 a | 0.14±0.004 a | 0.17±0.012 a | 4644±182 a*** | 3259±190 a | 516±19 a*** | 352±19 a | 6±0.2 a*** | 3.9±0.2 a |
| WS | 1818±51 b*** | 1151±51 b | 268±12 b*** | 175±8 b | 0.15±0.005 a | 0.15±0.004 a | 3465±60 b*** | 2677±98 b | 396±6 b*** | 293±12 b | 4.7±0.1 b*** | 3.3±0.2 b |

Plants were grown in pots with potting mix substrate for 28 Dat under well-watered (WW) and water stress (WS) conditions. Data shown are means ± SE (n=6). Asterisks indicate the significant differences between the cultivars and treatments, as determined by Student’s t-test: For these assays, significance differences are indicated as follows: * *P* < 0.05; ** *P* < 0.01; *** *P* < 0.001. Different lowercase letters indicate significant differences (*P* < 0.05) between well-watered (WW) and water stress (WS) treatments, in the same genotype, as determined by Tukey’s HSD tests.
